# Total hip arthroplasty for Crowe type IV developmental dysplasia of the hip combined with intertrochanteric fracture: a case report and literature review

**DOI:** 10.1186/s12893-020-00941-2

**Published:** 2020-11-11

**Authors:** Wei Chen, Yong Ma, Hui Ma, Mao Nie

**Affiliations:** 1 Department of Orthopaedic Surgery, People’s Hospital of Fengjie County, Chongqing, 404600 People’s Republic of China; 2 Department of Orthopaedic Surgery, The Second Affiliated Hospital of Chongqing Medical University, 76 Linjiang Road, Yuzhong District, Chongqing, 400010 People’s Republic of China

**Keywords:** Developmental dysplasia of the hip, Intertrochanteric fracture, Total hip arthroplasty

## Abstract

**Background:**

Total hip arthroplasty for Crowe type IV developmental dysplasia of the hip (DDH) is a complex procedure. Crowe type IV DDH combined with intertrochanteric frature is very rare.

**Case presentation:**

A 75-year-old patient suffering from left hip pain after a fall was sent to our hospital. Plain radiographs and computed tomography scans were used to diagnose this patient with DDH combined with an intertrochanteric fracture. We conducted a total hip arthroplasty using an S-rom prosthesis following subtrochanteric shortening osteotomy in this patient, after which steel wires were used to fix the intertrochanteric fracture. The patient did not suffer any significant intraoperative or postoperative complications, and treatment was sufficient to overcome lower leg abnormalities. The patient was encouraged to resume walking with support at 3 days post-surgery, and at 6-month postoperatively he had regained the majority of his original range of motion. At 10-month postoperatively, the intertrochanteric fracture and subtrochanteric osteotomy of left femur had healed effectively,and the patient’s VAS and mHSS scores had improved significantly.

**Conclusions:**

Total hip arthroplasty is an effective approach to treat patients suffering from Crowe type IV DDH combined with an intertrochanteric fracture, and can achieve satisfactory clinical outcomes.

## Background

Crowe type IV developmental dysplasia of the hip (DDH) is a severe deformity that results in hip dysfunction and an abnormal gait [1]. This condition is typically treated via total hip arthroplasty (THA), which is a technically demanding procedure. Intertrochanteric hip fractures commonly occur among older adults. However, there have not been any reports regarding the appropriate treatment of patients simultaneously suffering from both DDH and an intertrochanteric hip fracture. In the present report, we describe a rare case of a patient that presented with type IV DDH combined with an intertrochanteric fracture. This patient achieved good clinical outcomes following treatment via THA with subtrochanteric shortening osteotomy and greater trochanter fixation.

## Case presentation

**Fig. 1:**
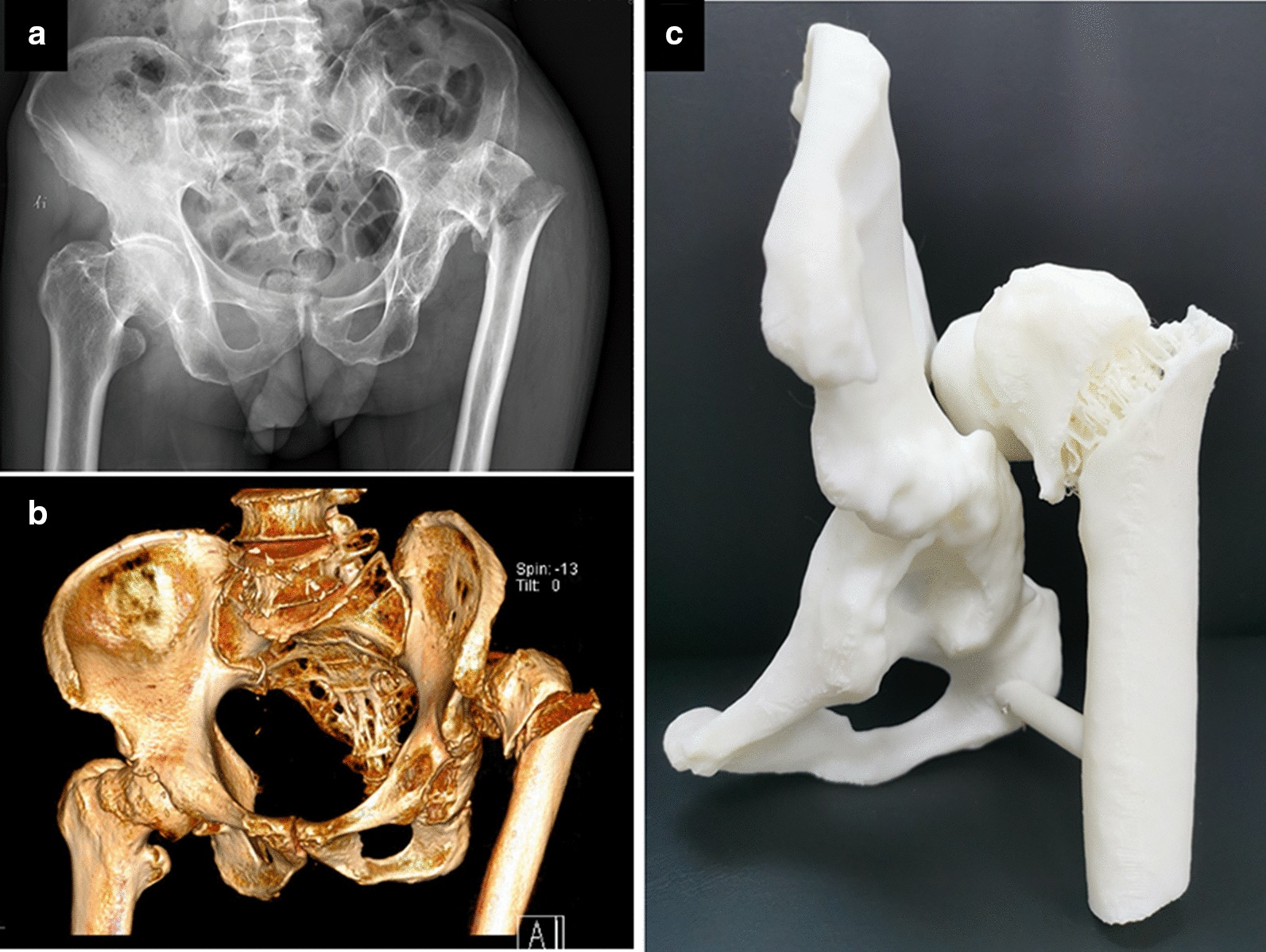
**a** Preoperative anteroposterior radiographs of the pelvis and hip. **b** Preoperative 3D computed tomography reconstruction model of the pelvis and hip. **c** Preoperative 3D-printed model of the pelvis and hip using the computed tomography data

A 75-year-old male patient was sent to our hospital suffering from left hip pain sustainedly after an accidental fall. Radiographic imaging revealed that this patient was affected by Crowe type IV DDH combined with an Evans type III intertrochanteric fracture of left femur (Fig. 1a). The patient reported a history of left hip trauma at the age of 10, at which time he did not accept proper treatment. The resultant left hip deformities had since caused the patient to walk with a limp. Prior to his fall, the patient was able to bear weight and work with moderate left hip pain. Physical examination revealed the left leg to be 4 cm shorter than the right leg, with local tenderness being detected in the left hip. In addition, percussive pain in the left femur in the axial direction and pain with internal and external rotation were noted. As passive activity caused serious pain, we were unable to measure the degrees of left hip motion. The patient had been diagnosed with hypertension for six years, and regularly took blood pressure control medications. Computed tomography scans revealed the high posterior dislocation of the left femoral head up to the level of the greater sciatic foramen(Fig. 1b). The original acetabulum was smaller and shallower (Fig. 1b). In order to better preoperatively evaluate the acetabulum and femur deformities in this patient, a 3D printed model was constructed (Fig. 1c).

The patient was offered two surgical options: THA or internal fixation to treat only the intertrochanteric fracture. After careful consideration and discussions with family members and the surgeon, the patient selected to undergo THA, and also provided informed consent for the publication of his case, which has not been reported previously to our knowledge.

Under general anesthesia, the patient was placed in the lateral decubitus position. The operation was performed via a posterior approach with an incision length of almost 20 cm. The external rotators were first detached, and then the femur neck was removed. After resection of the elongated hypertrophic joint capsule, the original acetabulum was clearly exposed and gradually reamed to 50 mm. Next, the 50 mm acetabular cup and a 28 mm polyethylene liner were placed in an appropriate anatomic arrangement with three screws. To clearly expose the proximal femoral canal, the femoral great trochanter fragment was overturned along the fracture line. Approximately 2 cm below the lesser trochanter, a 4 cm-long femoral shortening transverse osteotomy was performed by resecting the femur in accordance with preoperative planning. A modular S-rom femur stem was then installed from the level of intertrochanteric fracture end into the canal, and the femur was then de-rotated and the resected cylindric bone segment was cut longitudinally, after which these two pieces were bound onto the osteotomy site with wires. Equipped with 32 mm short metal head, the stem was easily reduced, after which the intertrochanteric fracture was reduced and stably fixed with steel wires.

**Fig. 2:**
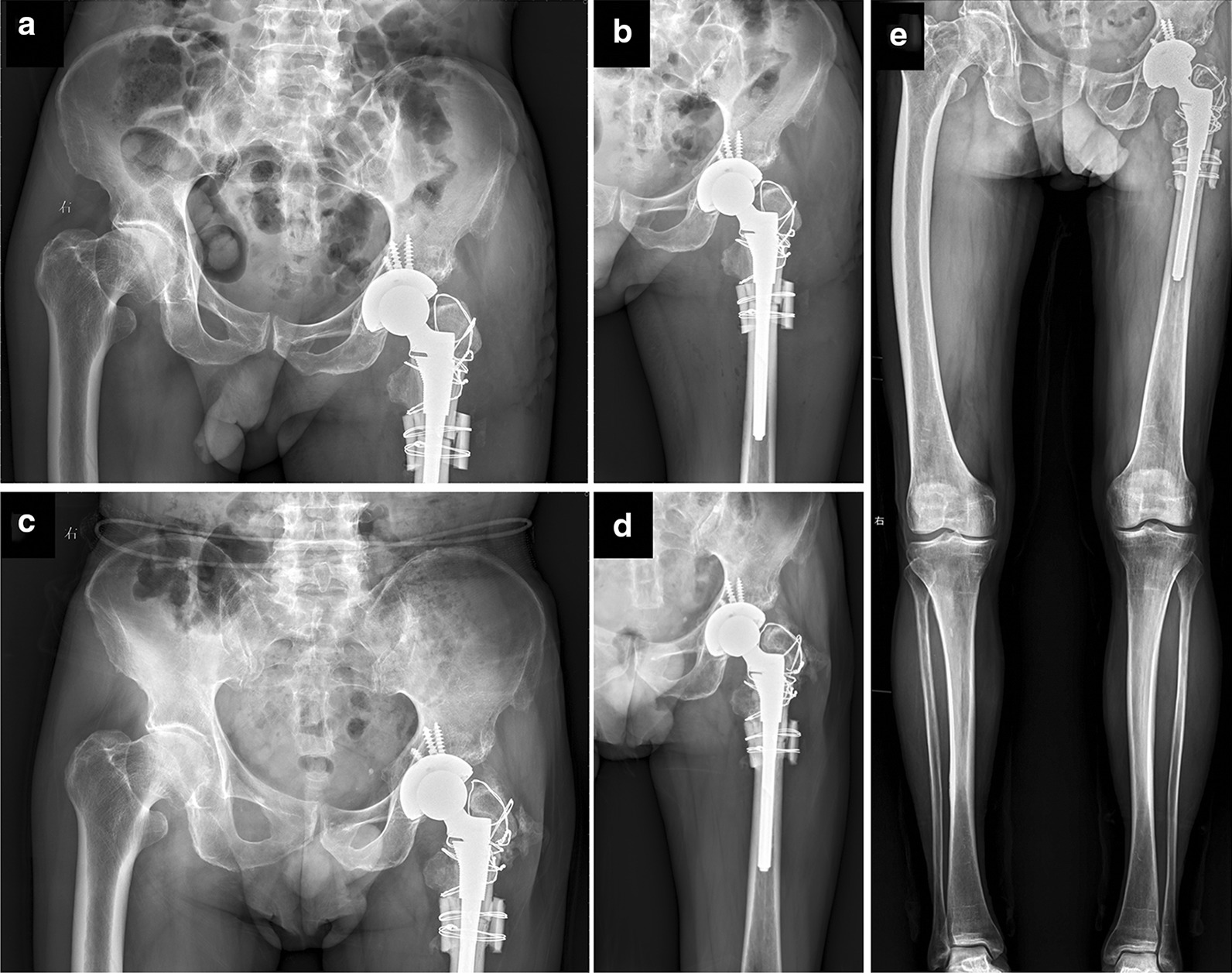
Anteroposterior radiographs of the pelvis and left hip joint at 3-day postoperatively (**a** and **b**) and 10-month follow-up postoperatively (**c** and **d**). **e** Full length weight bearing radiograph of lower extremities at 10-month follow-up postoperatively

**Fig. 3:**
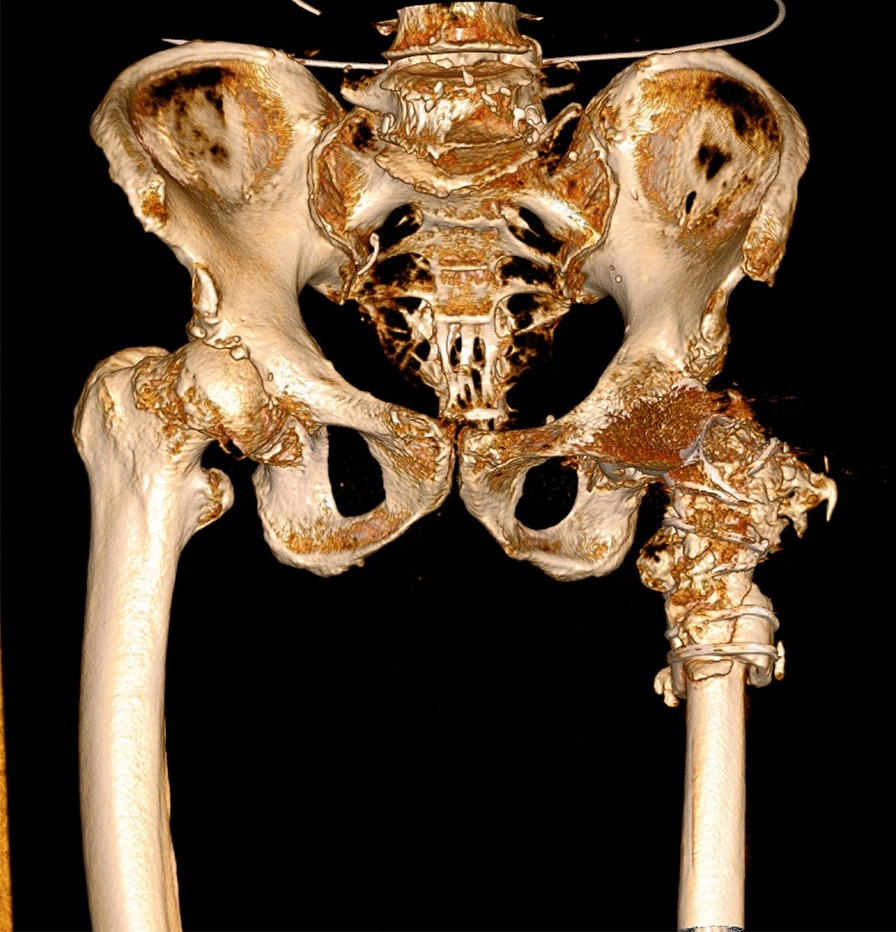
3D computed tomography reconstruction model of the pelvis and hip at 10-month follow-up postoperatively

Postoperatively, the patient was administered intravenous antibiotics and prophylactic anti-thrombotic treatment. Three days postoperatively, the patient was encouraged to stand and to walk with the aid of a walker. Postoperative plain radiographic images revealed that the original rotational center of the hip was restored and that the inclination and anteversion of the cup had been restored (Fig. 2a, b). The patient completed baseline visual analog scale (VAS) score and modified Harris hip score (mHHS) assessments via retrospective questionnaire, and also completed these assessments at 3 and 6 and 10 months postoperatively. Both VAS and mHHS scores were significantly improved at these postoperative follow-up time points (Table 1). Radiographic and computed tomography scans conducted upon most recent follow-up revealed that the prosthesis was properly positioned and the intertrochanteric fracture and subtrochanteric osteotomy had healed effectively (Figs. 2c, d, 3). Leg length discrepancy (LLD) measurements through the full length weight bearing radiograph of lower extremities showed the left limb to be 1 cm shorter than the right limb(Fig. 2e). Even though there was a little heterotopic ossification occurred on greater trochanter of left femur,the patient almost felt no pain and could walk up and down stairs without any assistance.

**Table 1:** Patient clinical scores

	Before injury	3 months postoperatively	6 months postoperatively	10 months postoperatively
VAS	5	1	0	0
mHHS	53	73	84	87

## Discussion and conclusions

DDH is a common disorder in infants and children. Early detection and non-operative management has been shown to be effective for the management of pediatric DDH [2]. When such non-operative treatments fail, surgical approaches are recommended. With increasing age, the associated deformities can become more complex, necessitating increasingly aggressive treatments including open reduction and femoral or pelvic osteotomy [3]. For symptomatic DDH in young adults, joint-preserving surgeries such as hip arthroscopy, periacetabular osteotomy (PAO), femoral head-neck junction osteochondroplasty combined with PAO, intertrochanteric osteotomy, or capsular arthroplasty are recommended to patients with minimal articular cartilage degeneration, as they could reduce or delay the degeneration of the hip joint [4]. A recent systematic review revealed that hip arthroscopy for borderline DDH with capsular plication improved short-term patient-reported outcome measures [5].

Crowe type IV DDH is a severe deformity that results from the long-term dislocation of the femoral head, leading to femoral head deformities, a high hip center, a short femoral neck with excessive anteversion, a narrow femoral canal, a hypoplastic true acetabulum with reduced depth and deficient superior bone stock, a posteriorly located greater trochanter, and soft tissue contractures [6]. Given that this condition can result in gait abnormalities and hip dysfunction, treatments primarily aim to achieve anatomic hip reconstruction in order to correct lower limb discrepancies, thereby increasing the odds of achieving satisfactory functional outcomes. THA is the most common treatment for DDH in adults [7]. However, the rates of complications associated with this treatment approach are significantly higher than those associated with other forms of primary THA [8–10].

With respect to the surgical technique, the acetabular cup should be placed into the true acetabulum, and a modular femoral prosthesis is recommended for the deformed medullary cavity. A number of different osteotomy approaches including greater trochanter osteotomy, lesser trochanter osteotomy, and subtrochanteric osteotomy are available as a means of achieving reduction and eliminating gait abnormalities while minimizing the risk of nerve complications. Of these osteotomy techniques, transverse osteotomy is the simplest and reliably achieves positive outcomes [6, 8, 9, 11, 12]. Common complications following THA for the treatment of Crowe Type IV DDH include LLD, intraoperative fracture, osteotomy nonunion, and nerve injuries, making this procedure more challenging to conduct [13]. Preoperative individualized measurement and osteotomy calculations are important steps to reducing LLD. Physical therapy and shoe lifts can be utilized to address LLD > 5–10 mm after THA. Internal fixation is a common means of addressing intraoperative fracture. As the sciatic nerve should be carefully dissected and palpated during operation, osteotomy is an effective method to avoid any sciatic nerve injury. To reduce sciatic nerve tension, the ipsilateral knee should be maintained in flexion when the limb is lengthened [13].

Intertrochanteric hip fractures are among the most common fractures affecting the elderly, and are primarily treated via internal fixation with intramedullary fixation. However, owing to the relatively significant failure rates of internal fixation and the advantages of arthroplasty, certain surgeons conduct primary hip arthroplasty for the treatment of unstable intertrochanteric hip fractures and achieve positive outcomes [14–16]. Nie et al. [17] conducted a meta-analysis comparing intramedullary fixation and arthroplasty as approaches to the treatment of intertrochanteric hip fractures in the elderly, and they found that arthroplasty was associated with lower rates of reoperation and implant-associated complications. However, intramedullary fixation was associated with reduced blood loss, lower transfusion requirements, shorter operative duration, higher Harris hip scores, and lower 1-year mortality rates.

Given the special circumstances associated with the present case, the internal fixation for treatment of intertrochanteric fractures could not relieve the symptoms caused by Crowe IV DDH.Total hip arthroplasty could be a better choice for this patient and it was not difficult to deal with the intertrochanteric fracture at the same time. Subtrochanteric shortening osteotomy was a safe way to correct the deformity of femur and facilitate reduction.The modular S-rom stem could provide secure fixation and stability for load bearing early. The patient did not suffer from any severe intraoperative or postoperative complications, and follow-up conducted at 10 months post-surgery indicated that his pain had been relieved and that his hip function was acceptable. The patient was satisfied with the treatment and is still undergoing regular follow-up. Overall, this case report is intended to provide orthopedic surgeons with an option to treat Crowe IV DDH combined with intertrochanteric fracture.

## Data Availability

All data are included in the section of Case Presentation and are available from the corresponding author on reasonable request.

## Abbreviations

DDH: Developmental dysplasia of the hip
THA: Total hip arthroplasty
LLD: Leg length discrepancy
VAS: Visual analog scale
mHHS: Modified Harris hip score
PAO: Periacetabular osteotomy
